# Tiny Lungs, Big Challenge: The Treatment Landscape of Pediatric Tuberculosis

**DOI:** 10.1155/pm/9686798

**Published:** 2026-10-28

**Authors:** Lucia N. Dumeni, Pradeep Kumar, Yahya E. Choonara

**Affiliations:** ^1^ Wits Advanced Drug Delivery Platform Research Unit Department of Pharmacy and Pharmacology School of Therapeutic Sciences Faculty of Health Sciences University of the Witwatersrand Johannesburg South Africa wits.ac.za; ^2^ Infectious Diseases and Oncology Research Institute (IDORI) Faculty of Health Sciences University of the Witwatersrand Johannesburg South Africa wits.ac.za

**Keywords:** child-friendly formulations, clinical outcome, multidrug-resistant TB, pharmacokinetics

## Abstract

Tuberculosis was a lethal disease until the early 1950s, when the first anti‐TB drugs were discovered. These drugs saved many lives until bacteria developed resistance in the 1980s. As a result, TB has re‐emerged as a global health challenge, affecting both drug‐susceptible and drug‐resistant cases. Although tuberculosis is a preventable and curable disease, it remains a major global health burden for both adults and children. High‐prevalence cases in children threaten eradication efforts because they can become fatal in the future. The review is aimed at compiling knowledge on pediatric TB treatment, particularly oral formulations. Efforts have been made over the years to improve pediatric TB treatment. Challenges remain in gathering pharmacokinetic data for children, distributing child‐friendly formulations worldwide, and strengthening collaboration among stakeholders. These issues affect clinical outcomes and hinder the eradication of this infection. Currently, there is a global initiative to improve access to high‐quality TB medications and to utilize new technology, such as developing nanoformulations and dispersible tablets, for better drug delivery. Continued progress, innovation, and fostering partnerships are necessary to overcome existing challenges in TB treatment and to develop child‐friendly formulations.

## 1. Introduction

### 1.1. Background

Despite tuberculosis (TB) being a preventable and curable disease, it continues to be a global burden, and children are also affected by this infectious disease. Pediatric TB has been reported to account for 12% of the global TB burden, which is about 1.25 million annually, but 17% of TB‐related deaths were recorded in children, with higher mortality recorded in children under the age of 5 years [1]. Underdiagnosed and undertreated cases hinder TB in children due to their nonspecific symptoms and diagnostic limitations [2].

Multidrug‐resistant tuberculosis (MDR‐TB) is also a major public health challenge, affecting global health security, economic burden, and access to high‐quality care [3]. Among the pediatric population with TB, 3% have MDR‐TB, accounting for around 25,000–32,000 cases per year; however, only 3%–4% get diagnosed and treated; unfortunately, 21% of MDR‐TB pediatric cases die [2]. Children are the most vulnerable population, and these high rates of MDR‐TB serve as a reservoir for future fatal cases, having a catastrophic effect on their physical health, impacting their mental health, and jeopardizing educated citizens′ academic careers [2]. Therefore, Figure 1 gives a summary of the gaps between children with MDR‐TB, those diagnosed, and those who receive the treatment.

**Figure 1:**
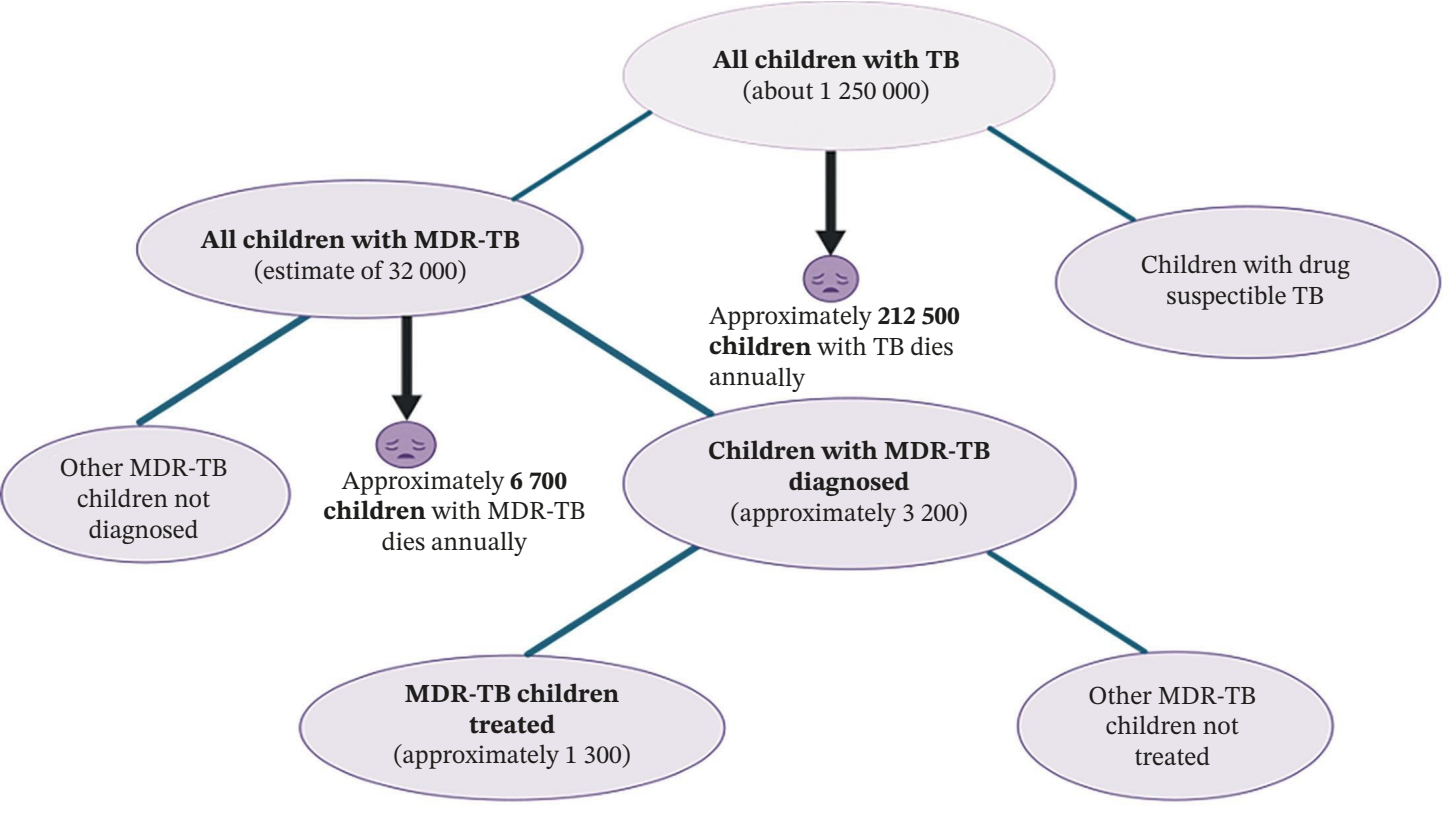
Statistical differences in detecting, diagnosing, and treating MDR‐TB in children per year. It indicates that over 32,000 children with MDR‐TB, only approximately 3200 children are diagnosed with MDR‐TB, and only around 1300 children are treated.

### 1.2. Problem Statement

The scarcity of age‐appropriate formulations for orally administered pediatric medicines is a critical, ongoing issue that requires urgent solutions across pediatric therapies [4]. Similarly, it is crucial to have a child‐friendly anti‐TB formulation for both drug‐susceptible tuberculosis (DS‐TB) and drug‐resistant tuberculosis (DR‐TB) to ensure correct dosing, reduce the risk of toxicity, and improve adherence through better acceptance for both children and caregivers [5]. Therefore, this study is significant because it addresses how pediatric TB has evolved over time, what known challenges exist, and the progress that has been made over the years to solve the problem.

### 1.3. Aim and Objectives

This review is aimed at assessing the current state of knowledge on pediatric TB treatment and the evolution of TB treatment over the years in both DS‐TB and DR‐TB.

The study objectives are as follows:

• To discuss, review, and synthesize the knowledge regarding current oral TB treatment for children.
• To understand current treatment challenges and proposed solutions to improve treatment outcomes by comparing the effectiveness, safety, and toxicity of medicines on the market.
• To assess advances and innovations made over the years in improving child‐friendly TB formulation.

## 2. Method

The keywords pediatric TB, drug‐resistant TB in children, TB oral formulations in children, and child‐friendly formulations were used alone and/in combination to search in Google Scholar, PubMed, and the Wits Library. No time limits were used because the study is aimed at assessing changes in treatment and any advancements made over the years. The review′s objectives are to make recommendations about enhancing oral pediatric TB care; no analysis of adult‐related data, other drug routes of administration, or non‐TB medications was conducted. The study included research papers on pediatric TB treatment that focus only on oral formulations, with nothing on injectable formulations.

## 3. Current Treatment Regimens

### 3.1. Emerging of MDR‐TB and Its Pathophysiology

*Mycobacterium tuberculosis* (M.tb) causes TB, and the strain that responds to first‐line drugs is referred to as DS‐TB, which was a lethal infectious disease until the 1950s. The scene shifted with the release of the first anti‐TB medications, para‐aminosalicylic (PAS), and streptomycin. Following these, new drugs such as pyrazinamide (Z), isoniazid, ethionamide, cycloserine (Cs), rifampicin, and ethambutol were launched, revolutionizing TB treatment and lowering the clinical result of fatal cases from 27% to 7% [6]. However, in the 1980s, some instances became exceedingly drug‐resistant and could not be treated with these treatments. This revival resulted in TB re‐emerging as a serious infectious disease, especially when M.tb is resistant to both rifampicin and isoniazid, known as MDR‐TB [1].

The goal of eradicating this fatal illness has been threatened by medication resistance in children, inadequate reporting, poor surveillance, a lack of children′s screening instruments, restricted hospital access, insufficient knowledge, and decreased financing for TB initiatives. Since children have more success stories with drug‐resistant therapy than adults, these barriers to TB treatment participation in this age range are impeding the efforts to eradicate this sickness [2].

Numerous research studies have investigated the disease′s spread to facilitate the development of targeted treatments. Tubercle bacilli in droplets enter the lungs′ alveoli during breathing and induce infection through aerosolization, according to [7]. Alveolar macrophages consume these bacteria, which are frequently killed or suppressed. In macrophages, M.tb reproduces intracellularly, leading to asymmetric cell division. The immune system may respond as the germs travel to the body′s bone structures via the lymphatic or circulatory systems. A granuloma is a bacterial jail that forms when a bacterium is isolated beneath immune cells. There are two types: primary and secondary TB. Primary TB is when someone is initially infected, and secondary TB is when there is a reactivation of previously contracted TB.

Therefore, Figure 2 illustrates how one can be infected with TB, which may initially be resistant to TB drugs, or how someone develops resistance while on medication. (a) There is an inhalation of M.tb droplets into the lungs after talking or coughing of someone with active TB. (b) The alveolar macrophage recognizes a threat; hence, it engulfs and digests the M.tb in an attempt to eliminate it. (c) After the T‐helper response, the immune cells produce a granuloma that will lead to (e) active disease if the immune system is weak or (d) some granulomas undergo caseous necrosis and lead to (f) latent infection because the immune system is strong. (g) When the immune system of the individual weakens, reactivation of this leads to (e) active disease. It is at this stage that, if the case advances, it can be transmissible and the cycle continues.

**Figure 2:**
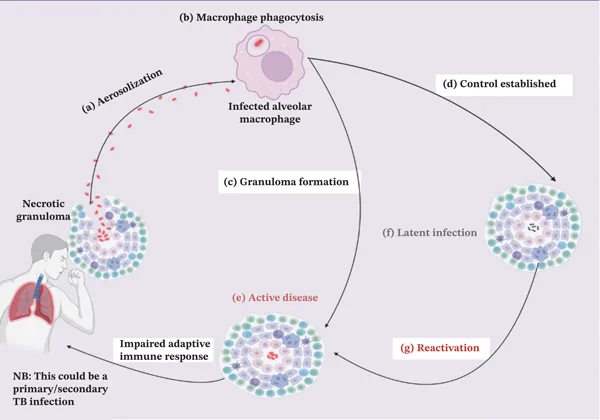
The pathophysiology of active tuberculosis (graphics prepared using Biorender′s default functions). The process starts from (a) aerosolization, (b) macrophage phagocytosis, and (c) granuloma formation and becomes (e) active disease or the immune system (d) established control, which turns into (d) latent infection, which only (g) reactivates when the immune system weakens to turns into (e) active disease again that can be infectious when an infected person talks or coughs and the process starts over again.

In addition, when the immune system cannot stop TB germs from multiplying, TB becomes active and children with active TB feel sick. The child may present with cough, reduced playfulness, weight loss, fever, and night sweats. TB disease commonly occurs in the lung, which is referred to as pulmonary TB, but it can also affect other parts of the body, referred to as extrapulmonary TB [1].

Diagnosing TB in children depends on assessment of a combination of patient history based on recent exposure and TB symptoms experienced, clinical examination including growth assessment, HIV status, chest x‐ray (CXR)/diagnostic testing if available, and other investigations based on site of infection (1). These factors are considered during TB testing and diagnosis in children and play a major role in following the treatment algorithm, which helps guide decisions about regimen selection, as illustrated in Figure 3.

**Figure 3:**
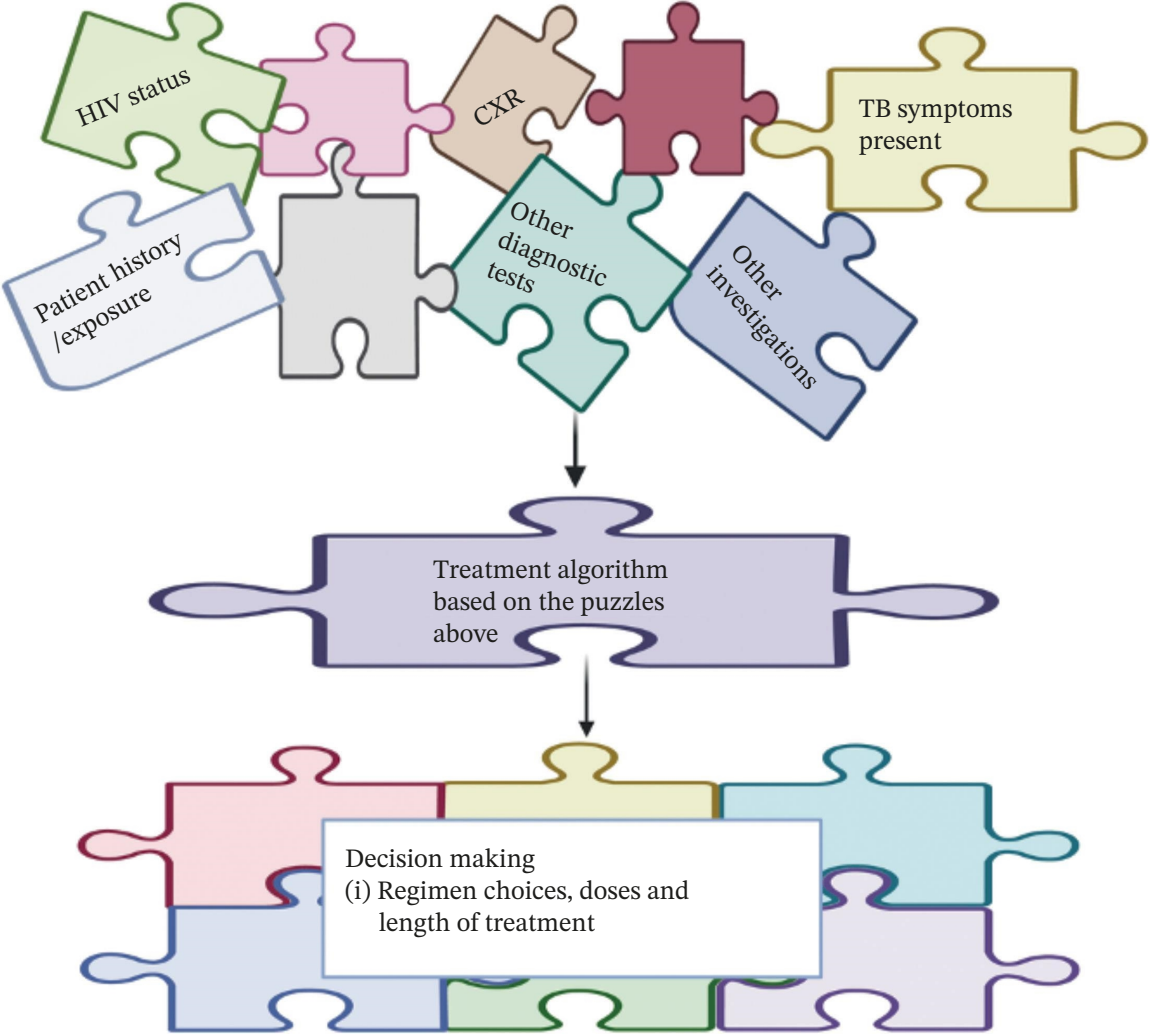
Building the TB “diagnostic puzzle” in children. The top blocks represent what needs to be assessed at diagnosis, which helps individualize treatment for every child with TB.

### 3.2. DS‐TB

DS‐TB has been treated with the standard 6‐month regimen for the past 50 years because several clinical trials conducted between 1946 and 1986 by the British Medical Research Council showed positive results. Based on those trials, DS‐TB can be cured with an initial 2‐month intensive phase using rifampicin, isoniazid, and Z, followed by a 4‐month continuation phase with rifampicin and isoniazid [8]. Therefore, the current WHO‐recommended treatment regimen lasts 6 months, comprising a 2‐month initial phase in which children receive isoniazid, rifampicin, Z, and ethambutol (HRZE), followed by a 4‐month continuation phase of isoniazid and rifampicin (4HR) [1].

Although the standard 6‐month regimen is widely adopted globally, researchers continue to work on developing a shorter regimen. Among the trials, Study 31/A5349 demonstrated that rifapentine combined with isoniazid, Z, and moxifloxacin can be used for a 4‐month therapy [9]. Therefore, the WHO recommended this regimen for persons over 12 years old for the treatment of pulmonary DS‐TB in May 2022 [10]. There is also an acknowledgement of a 4‐month shorter regimen for nonsevere TB in children, based on recent WHO trial data: 2 months of HRZ(E) and 2 months of HR [11]. Treatment in children is based on weight, with some references guiding the recommended dose for children weighing below 25 kg. Table 1 illustrates pulmonary TB treatment according to the child′s age and disease severity.

**Table 1:** Current WHO recommended regimens for drug‐susceptible pulmonary tuberculosis (PTB) in children based on age group and disease severity [1].

Age and severity of TB	Duration and composition of treatment regimen	
	Intensive phase	Continuation phase
Infants aged < 3 months or weighing < 3 kg		
PTB of any severity	Two months of isoniazid, rifampicin, and pyrazinamide (2HRZ) or 2 months of isoniazid, rifampicin, pyrazinamide, and ethambutol (2HRZE)	Four months of isoniazid and rifampicin (4HR)
Children and adolescents aged 3 months to < 12 years		
Nonsevere PTB	2HRZ or 2HRZE	Two months of isoniazid and rifampicin (2HR)
Severe PTB	2HRZE	4HR
Adolescents aged 12 to < 16 years		
Nonsevere PTB	2HRZ or 2HRZE	2HR
Severe PTB	2HRZE	4HR
PTB of any severity	Two months of isoniazid, rifampicin, pyrazinamide, and moxifloxacin (2HPZM)	Two months of isoniazid, rifampicin, and moxifloxacin (2HPM)
Adolescents aged 16 to < 20 years		
PTB of any severity	2HRZE	4HR
PTB of any severity	2HPZM	2HPM

Children enrolled in TB treatment are monitored to determine whether TB symptoms are resolving, whether side effects are being experienced, and for any other symptoms during treatment, while measuring their weight and ensuring treatment adherence. Sputum samples should be repeated at 2 months after the start of treatment, and for those with unconfirmed TB, the sample should be repeated at 2 months after the start of treatment, and the CXR does not need to be repeated if there is a good treatment response [10].

According to WHO, HIV‐negative children will be followed up 2 weeks after the start of treatment, then at 4 weeks, followed by 2 months after the intensive phase and every 2 months until completion, either 4 or 6 months of treatment. HIV‐positive children will be followed up similarly until the intensive phase, but will be followed up monthly until the treatment completion. Clinical monitoring is done at the end of treatment duration and determines if the child is successfully treated [10].

Medication counselling is very important, with emphasis on adherence to TB treatment. This is because if treatment is stopped, the children might get sick again and the likelihood of TB strains developing resistance increases. Therefore, to curb the possibility of drug resistance, directly observed therapy (DOT) was introduced to optimize TB treatment monitoring. In this case, healthcare workers visit each child on treatment to observe that they are taking their medication and that treatment is working as intended without side effects [12].

### 3.3. DR‐TB

Following positive screening and diagnosis, it is recommended that children with MDR‐TB receive second‐line treatment based on bacteriological confirmation and risk factors. It suggests utilizing child‐friendly second‐line medications, avoiding injectables, and taking baseline weight and height measurements. Regularly evaluate the clinical, radiological, and microbiological response to treatment to minimize unnecessary hospitalization and disruption of education. It also focuses on monitoring for adverse events(AEs) and adherence, as well as providing age‐appropriate social support during screening, diagnosis, therapy initiation, and follow‐up [1].

MDR‐TB treatment in children mainly includes at least four effective drugs, prioritizing WHO Group A and B drugs (1). The WHO recommends injectable‐free regimens for children and divides drugs into three categories: Group A includes levofloxacin (Lfx)/moxifloxacin, bedaquiline (BDQ), and linezolid (Lzd); Group B has clofazimine (Cfz) and Cs; and Group C includes delamanid, ethambutol, Z, ethionamide, PAS acid, and amikacin. Since 2022, BDQ and delamanid have also been recommended for use in children [1]. Table 2 summarizes and supplements the explanation above based on TB resistance strains.

**Table 2:** Possible individualized multidrug‐resistant and rifampicin‐resistant TB treatment regimens for children according to the WHO recommendation [1].

Fluoroquinolone susceptibility	Regimens	Additional medicines
Fluoroquinolone‐susceptible	Bedaquiline–levofloxacin–linezolid–clofazimine‐(cycloserine)–(BDQ‐LFX‐LZD‐CFZ‐Cs) (for 6 months)	Cycloserine, delamanid, P‐aminosalicylic acid, ethionamide (ethambutol, pyrazinamide)–Cs, DLM, PAS, ETO (EMB, PZA)
Fluoroquinolone‐resistant	Betaquiline–linezolid–clofazimine–cycloserine–(delamanid)–BDQ‐LZD‐CFZ‐Cs‐(DLM) (for 9 months)	DLM, PAS, ETO (EMB, PZA)
Fluoroquinolone‐resistant and BDQ (±CFZ)‐resistant	Linezolid–cycloserine–delamanid–ethambutol–pyrazinamide–(LZD‐Cs‐DLM‐EMB‐PZA) (for 9–12 months)	Meropenem in combination with clavulanic acid, ethionamide (ETO), P‐aminosalicylic acid (PAS)

## 4. Challenges in Pediatric TB Treatment

### 4.1. Pharmacokinetic (PK) Study

There is little information on children′s formulation, and most of the treatment guidelines use pediatric data extrapolation from adult data. Therefore, it is recommended to explore the PK of different TB drugs in the children population from all corners of the world to improve their treatment outcomes [13].

Most studies on safety and tolerability have cautioned against the use of these TB medications. The study that explores the safety and tolerability of moxifloxacin in children has recommended its use, but with proper monitoring of side effects, especially when used with other drugs [14]. While the study found that the current recommended dose of moxifloxacin in children, 10–15 mg/kg, was safe, the exposure target concentration was not reached. It was further recommended to consider the safety assessment in adjusting the dose, especially if other QT‐prolonging agents are coadministered [15].

PK studies in Ghanaian children receiving first‐line TB treatment have shown that the lowest target concentration was achieved at the WHO‐recommended dose of rifampicin and isoniazid, but the plasma target concentration was not achieved for Z and ethambutol [16]. In contrast, the updated WHO weight‐band dosing recommendations and first‐line fixed‐dose combination tablets for TB treatment in children achieve adequate plasma drug exposure. Despite adherence to WHO dosing guidelines, the study exhibits low Z and rifampicin plasma exposures and overexposure for isoniazid and ethambutol [17]. This was compared to the 95% confidence intervals of the global pediatric normalized for the WHO‐recommended dose (mg∗h/L): ethambutol (6.4–10.0), isoniazid (15.5–22.6), rifampicin (29.4–40.3), and Z (339.9–413.7); it further showed that most of the isoniazid and ethambutol area under the curve (AUC) values were above the WHO‐recommended dose, as opposed to the Z and rifampicin AUC values that were within or below. It also indicates that there was underdosing in children with a weight of 4–7.9 kg in rifampicin and Z, as their AUC values were below the recommended intervals [17].

The study of 51 children in Tanzania between January 2014 and January 2016 has also pointed out the limited PK studies in children treated with TB. Therefore, plasma concentrations of rifampicin, isoniazid, Z, and ethambutol were obtained from children at the end of the intensive phase of TB therapy, using the revised dosing recommended in Tanzania. Unfortunately, the target drug concentrations of rifampicin, isoniazid, and ethambutol were still not reached with the revised dosing [13].

The concentration of first‐line TB drugs at the site of infection was determined in children with intrathoracic TB. Although penetration characteristics are similar to those in adults, low plasma exposures in children lead to low site exposure for rifampicin, Z, and ethambutol, except for isoniazid. The results showed lower penetration of isoniazid, rifampicin, and Z in lymph nodes than in plasma and ethambutol accumulated in tissues [18].

Furthermore, the PK study on children treated with isoniazid and Z assessed the impact of age and nutritional status. In accordance with the revised WHO drug dosage, isoniazid was found to be adequate for appropriate age and nutritional status, but Z has shown significantly lower drug levels in children < 3 years and malnourished children [19].

The PK of isoniazid were assessed in children between 1 and 15 years old while comparing two recommended doses per day: 5 and 10 mg/kg/day. About 65% of children on 5 mg/kg/day have failed to reach the optimal therapeutic range, while those on 10 mg/kg/day have higher AUC and maximum concentration (Cmax) above the required range [20].

The PK study was done in children aged less than 18 years, where those < 12 years were treated with 20 mg dispersible BDQ tablets, while those > 12 years were treated with the available adult formulation. Within 96 weeks of follow‐up, BDQ showed comparative PK and safety profile to that of adults. The palatability of BDQ 20 mg was 82%. Few adverse events(AEs) were recorded that led to discontinuation of treatment and serious adverse events, but no deaths were recorded [21].

The efficacy and safety of an all‐oral BDQ containing regimen in a group of children aged 8–16 years was compared with a control group receiving an injection‐containing regimen (9–15 years) for pediatric patients with MDR‐TB in China. There was a significantly higher treatment outcome in children receiving an all‐oral BDQ‐containing regimen compared to the control group, with 100% versus 83.3% results, respectively. It took a shorter time of 4 weeks for sputum culture conversion in the BDQ group compared to 8 weeks in the control group, and the incidence of adverse events was significantly less in the BDQ group, with no adverse events (AEs) leading to treatment discontinuation compared to the control group (48.6% vs. 71.4%) [22].

In many instances, TB medication has been recommended for use in children without adequate dosing guidelines, limited safety, and no PK data. Therefore, the Cfz‐containing regimen in children < 18 years was analyzed using PK sampling and electrocardiograms at predose and at 1, 4, and 10 h postdose. A linear concentration‐QTcF relationship was observed with Cfz concentration associated with an increase in QTcT, but no severe prolongation [23].

The PK and pharmacodynamic studies were conducted in children undergoing second‐line TB treatment (some on kanamycin, fluoroquinolones, ethionamide, para‐amino PAS acid, and Cs). This study indicated that > 80% of children had successful treatment outcomes, and only a few with interindividual variability did not reach the target plasma concentration, suggesting further pharmacogenomics investigations [24].

The PK study on moxifloxacin 100 mg dispersible tablets and standard moxifloxacin 400 mg nondispersible adult formulation (crushed or suspended in water) in children aged < 15 years and weighing > 5 kg was assessed according to the WHO‐recommended dose. Dispersible formulations are favorable for younger children as they are easier to dose and administer. This study indicates that the recommended WHO dosing risks underdosing young children < 10 kg and optimizing the dose by 33%–56% to reach the adult reference exposure, but the simulations showed that the exposure was achieved in children > 10 kg [25].

A randomized Phase 3 trial done in children aged < 29 days to > 18 years with TB meningitis was conducted in Asia and Africa. The children were randomized in two groups receiving either a 24‐week intensified regimen (isoniazid 20 mg/kg, rifampicin 30 mg/kg, Z 40 mg/kg, and Lfx 20 mg/kg) or a 48‐week antituberculosis therapy (ATT) regimen and 8 weeks of high‐dose aspirin or placebo. Hence, the evaluation of both new anti‐inflammatory agents and optimized ATT regimens in children with TB meningitis is required. This SURE trial is expected to be completed by December 2025, but by June 2024, 369 children had been enrolled, with low loss to follow‐up [26].

Therefore, further studies on TB drugs with proper formulation and proven bioavailability in children are important steps to have maximum drug exposure. Other studies suggest that higher starting doses and peak concentrations might improve microbial activity, but finding the optimal doses and exposure levels to correctly estimate treatment duration and outcomes is equally important [13].

### 4.2. Clinical Outcomes

A retrospective study was conducted in Georgia between 2009 and 2011 to assess the characteristics and treatment of children diagnosed with MDR‐TB. It demonstrates a high rate of successful treatment outcomes in children, as proven in many studies around the world. They were treated with only six drug regimens, with an injectable and Lfx; most importantly, they have received educational talks, nutritional, and transportation support. However, they reported a high default rate (20%) [27].

An observational study done in Cape Town, South Africa, has shown a high treatment rate of > 90% in children below 15 years with MDR‐TB between January 2009 and December 2010. Although it is not possible to confirm MDR‐TB diagnosis in children, it should not be the cause of delay in the treatment. Further suggestion indicates that a reduction in treatment duration for nonsevere MDR‐TB could improve treatment outcomes [28].

A study to observe the treatment outcomes of Category II anti‐TB treatment in Indian children between 2004 and 2012 was reviewed. Category II anti‐TB was initiated due to worsening clinical conditions (36.8%) and relapses (36%), and 64% recorded treatment success, 16% of treatment failure, and 20% of default. The positive effectiveness of Category II anti‐TB in children encourages efforts to identify children who have failed and defaulted from previous regimens [29].

The study has evaluated three cases of MDR‐TB treatment in children, and they have affirmed that there are several limitations to the existing regimens. The existing treatment gap could be widened by the toxicity, formulation, and cost challenges of the available second‐line drugs (SLDs). Ultimately, achieving better medication for those children who need it most should be a global target [30].

The medical records and laboratory records of children with MDR‐TB under 13 years were examined to identify pretreatment loss and analyze demographic and clinical characteristics due to loss to follow‐up in three provinces in South Africa. The study in three provinces in South Africa found early loss to follow‐up and failure to initiate treatment in children compared to adults. Hence, the burden of MDR‐TB in children is underestimated and results in poor outcomes. There is a need for strong communication for referral and follow‐up on these diagnoses [31].

A case report described a 12‐year‐old who has been taking BDQ for 18 months despite it being recommended for 8 months only. It was unfortunately used as a lifesaving, and luckily, the treatment outcome was achieved without compromising the safety of this child. The child was diagnosed with TB in December 2018 and had treatment failure; thus, he was later diagnosed with MDR‐TB, which is only susceptible to amikacin, capreomycin, Cs, Lzd, and BDQ. Therefore, the child was started on amikacin, Cs, Lzd, and BDQ. After withdrawal of amikacin for 3 months in the treatment due to sensorineural hearing loss, the child continues Cs, Lzd, and BDQ. BDQ was not approved to be used by 18‐year‐olds nor for more than 8 months. With the father′s consent, it was used in this case, and no cardiotoxic effects were observed. This study has shown the effectiveness and safety of prolonged use of BDQ in children [32].

The study conducted in Dushanbe, Tajikistan, between January 1, 2013, and July 1, 2019, showed that introducing modified regimens for children with MDR‐TB yields positive treatment outcomes and reaffirms the continuous use of treatment regimens in other countries. In 2013, the national guidelines recommended only standard regimens for RR/MDR and individual regimens for RR/MDR and XDR. In April 2015, the standard regimen was discontinued, and BDQ and delamanid were introduced to the individual regimen for XDR. Fortunately, a short treatment regimen with injectables was introduced in December 2016 for RR/MDR‐TB, and a short regimen (STR) without injectables was introduced in 2019 [33].

A comparison between 4 months and 6 months of TB treatment in children has shown that both yield similar treatment outcomes, with minimal side effects and a lower treatment cost with 4 months of first‐line treatment [11].

A Phase I and II trial on the use of delamanid in children aged 0–17 years has shown a safety similar to that of adults and demonstrated a favorable treatment outcome after 24 months. Additionally, the child‐friendly formulation used in this study was found to be palatable by children, parents, and nurses [34]. Figure 4 shows that the new delamanid formulation was highly palatable, as observed from parent/patient self‐reports and clinician/nurse observations in children < 5 years old. For instance, in children between 3 and 5 years old, one child neither liked nor disliked the medication on Day 1 but, on Day 10, showed that the child liked the medication. While for < 2 years old, the nurses observed that there was a patient who disliked the medication on Day 1, but there was no dislike on Day 10.

**Figure 4:**
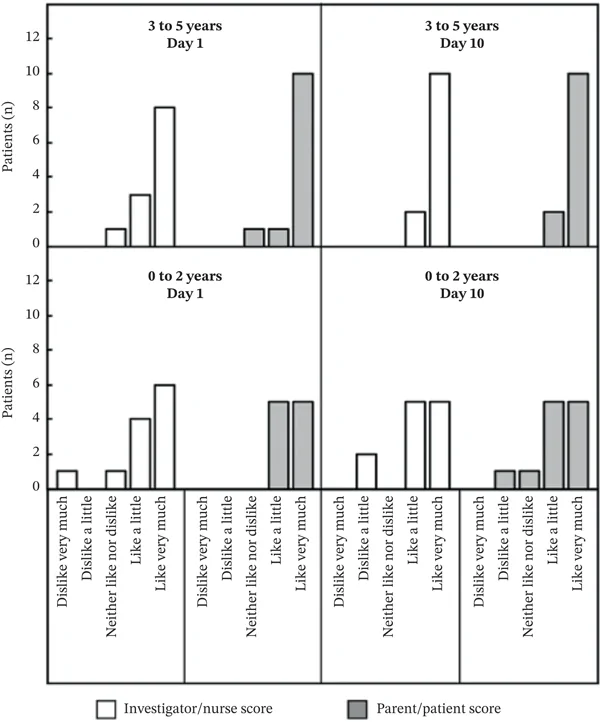
Assessment of delamanid pediatric formulation palatability from nurses and parents/patients′ perspectives. Reproduced from [34] under the terms of the Creative Commons Attribution 4.0 International license.

TB meningitis treatment needs serious intervention through timely diagnosis and prevention of neurological impairments, thus improving survival outcomes. The treatment has a high mortality rate of 42.5%, with 20% full treatment success and 36.25% of morbidity after treatment [35].

The first case of BDQ resistance in a 17‐year‐old boy is alarming, as the use of BDQ for all age groups is shorter, safer, and more effective. The boy was initially diagnosed with rifampicin‐resistant (RR) and developed BDQ resistance during treatment. He was given a shorter regimen of all drugs from Groups A (Lfx, Lzd, and BDQ) and B (Cfz and Cs) for 9–11 months; in the fourth month of treatment, the drug sensitive test (DST) had detected resistance to BDQ, and the treatment was declared failure and substituted into a longer term regimen consisting of Lfx, Lzd, Cfz, Cs, and Z for 18–24 months [36].

Despite the recommendation of 9 months of oral treatment, a study to assess treatment success and safety in children has shown good results and suggests considering the use of modified short treatment regimens (mSTRs) in programmatic conditions. High treatment success and good safety results were observed with mSTRs compared to 2020 WHO‐recommended STRs [37]. The treatment success rate in children is high, with about 94.9%, and most children enrolled on appropriate anti‐TB regimens get cured with no adverse effects. DOTS is a good strategy to ensure adherence [38].

The study compared the efficacy and safety of first‐line TB regimens with a first‐line regimen containing Lzd between January 1, 2016, and December 31, 2023, in the Department of Infectious Diseases, Children′s Hospital of Chongqing Medical University. It was found that the HRZEL regimen has similar efficacy as the conventional HRZE regimen in children with TB meningitis, but with additional adverse effects. Therefore, further evaluation of the use of Lzd and close monitoring of adverse effects are needed [39].

A retrospective study was done between June 2016 and May 2023 in Indonesia to assess the clinical features, adverse events, and treatment outcomes in children with MDR‐TB. The results indicate that most children were adolescents aged 10–17 years; successful treatment outcomes were 70%, 20%, died; and 10% were lost to follow‐up. The children who experienced Grade 1–2 adverse effects (AEs) were 53% and 5% had Grade 3–4 AEs that included hepatotoxicity, QT prolongation, hearing loss, and rash/hyperpigmentation. The results might reflect complex patient pathways limiting access to care [40].

A retrospective review conducted between 2014 and 2024 examined children between 2 and < 21 years old who are receiving once weekly isoniazid and rifapentine (3HP) under DOT in the United States. Over 94% of children have completed 3HP for TB infection, and the rest did not complete the treatment because of experiencing adverse events [41].

### 4.3. Formulation Gaps

Even though child‐friendly formulations are available in the market, they are not accessible to all the areas where there is a burden of MDR‐TB in children, particularly in developing countries, due to highly priced essential medications [42]. Therefore, the concept of dosage manipulation has been resorted to. According to [43], dosage manipulation is defined as all activities performed before administration to change the dosage formulation to make it suitable for pediatric patients. An observational study done in South Africa for MDR‐TB children inpatients has indicated that among eight identified drugs used for the treatment, only Lzd and PAS acid were registered as the child‐friendly formulations in South Africa. Hence, the remaining six drugs in capsule and tablet form were cut and dissolved in distilled water to obtain a suitable dose for pediatric use. The nurses indicated that it is time‐consuming and most drugs are unpalatable [43].

Additionally, some studies have developed standard formulas that can be used at a practical level. According to [44–46], extemporaneous preparations for the three main drugs used in MDR‐TB were formulated using simple syrup as a vehicle and using a method that can be done in the pharmacy. It verified how long this formulation remained stable, as well as its pH, potency, and microbial count, at different intervals within 30 days. Therefore, this is the adopted formula to prepare MDR‐TB oral treatment for pediatrics in different hospitals when child‐friendly formulations are not readily available.

The research on child‐friendly formulations available emphasizes compounding methods to be used in case medicines that are not available as dispersible tablets and which components to consider for a formulation to be optimal for children′s use. It also focused on the shortcomings of compounded medication because uniform particle sizes may cause dose differences, and other solvents need to be considered for insoluble drugs in water [47].

The acceptance of extemporaneous medication was assessed in [48], where children currently on MDR‐TB drugs were interviewed and it involved the caregivers′ and children′s opinions. This study has shown that the acceptability of current formulations remains very low and has consequences for both children′s treatment outcomes. They urge being involved in the early stages of new formulations that are sweeter, colorful, and easy to administer.

Access to antitubercular medications, particularly second‐line therapy, is a major challenge in the treatment of MDR‐TB in children. A survey of 22 countries found that nearly one‐third (36%) do not have access to BDQ or delamanid. Individual patients without treatment options are defined by country legislation and processes, as well as the product manufacturer. Early access is an ongoing process, and postmarketing safety monitoring can ease regulatory and provider worries. Drug request complexity, limited direct interaction with manufacturers, and insufficient administrative support all contribute to limited availability. The Stop TB Alliance provides a one‐stop bundled procurement and supply system for antitubercular drugs. Child‐friendly drugs are necessary to maintain the viability of approved pediatric dosages. The palatability of drugs is another relevant problem in the management of MDR‐TB in children, particularly for the youngest patients [49].

Furthermore, the evaluation of the current treatment of DR‐TB in children [50] showed that the main problem is with screening and diagnosing children. Despite a successful treatment rate, there is a need to detect more children. The choice of drug to be used and the length of treatment need to be considered depending on the disease severity, resistant drugs, and availability of a suitable formulation. Proper health education, medication counselling, support, and close clinical monitoring are essential to detect and manage adverse effects early, as they determine treatment adherence.

Extemporaneous preparation of moxifloxacin 20 mg/mL in a 1:1 mixture of Ora‐Plus and Ora‐Sweet/Ora‐Sweet SF has shown stability for 90 days when stored in the amber bottle at room temperature, and it also maintained 90% of drug content without microbial growth and change in color [51]. BDQ liquid formulation was prepared from 100 mg BDQ tablets (immediate release) to obtain 20 mg/mL in simple syrup and sugar‐free formulations using readily available and affordable ingredients. The main aim of the preparation was to provide a ready‐to‐use children′s formulation, unlike dispersible tablets that might be tiring to prepare and lead to medication wastage. The extemporaneous preparation in both syrup and sugar‐free forms was found to be suitable for use for 15 days if stored at room temperature. Though they were observed for 30 days, they were physically and chemically stable, but the United States Pharmacopoeia (USP) microbial count limits were at 15 days for syrup and 30 days for sugar‐free [44].

A simple field‐friendly formulation was used to prepare Cfz 10 mg/mL in syrup and sugar‐free ingredients. This formulation was then evaluated for appearance and pH, storage stability, microbial growth and potency, the tests also assessed the time taken to prepare a ready for dispensing bottle of this formulation. It only takes about 6 min to prepare in the pharmacy, making it reasonable for the patient′s waiting time. The formulations were stable for 15 days at room temperature and ready to use. It was found that organoleptic properties could be enhanced by using additional flavors and sweeteners, but considering the availability in the field and accessibility in the pharmacy [46].

Delamanid was prepared as a syrup and sugar‐free formulation to facilitate field‐friendly preparation in areas where dispersible medications are not yet accessible. It solved a problem of having a ready‐to‐administer formulation, but the syrup formulation can only be kept for 15 days, and the sugar‐free formulation can only be kept for 30 days. This might still be an issue for those who stay far from the hospitals, as they require travelling for a refill more often [45].

An observational study was done in South Africa to determine how DR‐TB drugs not available in a child‐friendly formulation are manipulated before being administered. The study focused on inpatient practice, and only Lzd and PAS out of eight medications were available as a suitable child formulation. Therefore, extemporaneous preparation was found to require time and delay administration and the instability of drugs [43].

Stability study for Cfz 10 mg/mL extemporaneous preparation from Ora‐Blend SF was analyzed in the study. It was found to be stable for 60 days after being kept at room temperature, retaining > 94% of drug content, and slightly degraded to 87.4% when kept at 4°C. There was no phase separation or color change in either preparation for 60 days [52].

The acceptability of Lfx 250 mg was evaluated compared to a placebo. It resulted in poor acceptance at Week 8 with 25.1% compared to 6.2% for those on placebo. After 24 24‐week courses, there was a slight improvement to 36.8% on Lfx and 12.9% on placebo. In this study, 11.7% of children swallowed 250 mg Lfx as a whole or in high doses at Week 8 and 34.4% at Week 24. Therefore, Lfx 250 mg can be used as an alternative to the dispersible formulation. LFX 250 mg can be used in children who can swallow a half/whole tablet as an alternative to dispersible Lfx 100 mg [53].

### 4.4. Lack of Collaboration

Even though DOT strategies have shown great results, there is a need to prioritize early diagnosis of pediatric cases with MDR‐TB instead of the current delay caused by reliance on the adult case definition. This will prevent progressive and chronic illness in children by earlier treatment initiation in all DOTS‐Plus programs [54]. Hence, the study assessed the impact of MDR‐TB and its treatment in children on individual, family and social levels. It was found that side effects of the medication cause significant physical, psychological, and academic challenges in children and hinder adherence to treatments, too. Notably, older children revealed that there is persisting internalized stigma, which is why caregivers suffer from treatment‐related financial and psychological costs. Therefore, there is a need for psychosocial support for both children and caregivers [55].

Healthcare providers at the frontline of Peru′s TB control demonstrated a knowledge gap in extrapulmonary disease, diagnosis, and prevention in childhood TB, which might delay or miss diagnosis and lose the opportunity for prevention. Therefore, educational intervention to improve childhood TB care and outcomes is highly recommended [56]. There were also assessment and diagnosis issues in children under 5 years due to poor team skills and understanding of the diagnostic algorithm [57]. The evaluation of the barrier to childhood TB care study′s findings urges the need for child‐friendly formulation, enhancing education on isoniazid preventive therapy (IPT) to both healthcare providers and parents and coming up with strategies to promote adherence to IPT so that it does not result in isoniazid resistance by providing support and supervision by health workers and lower dose regimens [58].

A study assessing the management of MDR‐TB in children by pediatricians in India indicates that management practices are still suboptimum, and there is an urgent need for inclusive and better engagement with the private sector to enhance TB management practices and immediate TB diagnosis that prevent drug resistance [59]. The assessment of the recently introduced fixed‐dose combination for the first‐line TB treatment in children is needed to understand how it is accepted by children and caregivers. Though it has an appealing orange color, there were still administrative challenges. Hence, it urges drug designers to involve caregivers in formulating a child‐friendly formulation to understand the practical administration aspect. At the moment, healthcare workers should support caregivers with the ingestion method and supplement educational intervention for better adherence and effectiveness [60].

Perspectives of parents on treatment success rate in children with TB have fostered the integrated approach needed and identified the motivational factors among parents of children with TB. The success rate is achieved through good support and trust from healthcare workers and psychosocial support for the caregiver so he/she remains determined throughout the treatment journey [61].

The study assessed the preference of administration, degree of compliance with label instructions and the accuracy of manipulation using different administration devices. Extemporaneous preparation of azithromycin with the instruction of shaking before use, different administration devices were used, and it was found that most volunteers did not comply with “shake before use” and used a double‐dosing spoon over an oral syringe and a transparent dosing spoon. The administration devices were found to be nonequivalent. There was an inaccuracy in using dosing spoons that might lead to underdosing or overdosing. Including the reconstitution cup in the package might increase the risk of incorrect use of administration devices, and oral syringes had the smallest deviation in measuring an accurate dose [62].

A qualitative study was done in adolescents (10–19) with MDR‐TB in Peru between August 2018 and May 2019 on their perspectives on adherence. The results indicate various barriers, most notably health‐based DOT, long treatment duration, adverse events, and symptom resolution. Therefore, caregiver support has helped overcome some of these challenges. It is important to evaluate DOT strategies, improve adolescents′ behavioral skills needed to adhere to treatment, and provide a strong support system for the caregivers [12].

The investigation of factors influencing compliance in children with TB between 2019 and 2021 in China has shown poor adherence in pediatric patients. The most influential factors were parents′ educational level, how many times anti‐TB medication is taken per day, and parents′ understanding of the disease. Therefore, the study recommends enforced health education for the parents to improve adherence to TB treatment [63]. Figure 5 illustrates the ideal stakeholders required in the pharmaceutical life cycle to enhance clinical outcomes, and each stakeholder contributes valuable insights to the development of better pediatric TB treatment formulations.

**Figure 5:**
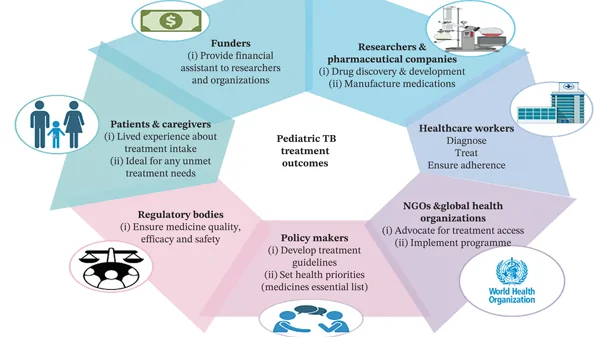
Stakeholders needed for an ideal collaboration to improve treatment outcomes in pediatric TB. This circular infographic summarizes key actors, their primary functions, and how each contributes to successful diagnosis, improved adherence, access, and treatment outcomes for children with TB. There is no hierarchy among stakeholders in the pharmaceutical cycle, and all contribute equally.

## 5. Recent Advancements and Innovations

### 5.1. Global Drug Facility (GDF)

The introduction of the Global TB Drug Facility on March 24, 2001, as a project of the Global Partnership to Stop TB was an initiative to improve access to high‐quality TB drugs while aiming to provide TB drug treatment to up to 11.6 million patients over 5 years and assist countries to reach the WHO global TB control targets by 2005. To date, GDF has created a flexible supply system to meet different aspects of the program, rapidly establish procedures, facilitate the reduction of TB drug prices, and assist in the expansion of DOTS in countries while setting product standards and maintaining collaboration with partners [64].

GDF was launched in 2001 as a response to some challenges in providing access to anti‐TB drugs by providing grant services to countries that lack financial means, by introducing direct procurement services to assist countries with ineffective procurement systems, and by providing technical support services to empower the management and monitoring of anti‐TB drugs and improve the quality of TB DRUGS. In 2006, GDF started providing pediatric anti‐TB. This aims to assure availability of high‐quality, appropriate formulation and affordable anti‐TB drugs to children [65].

There is a solution to the long‐awaited distribution of available simple, quality‐assured, and child‐friendly formulations in correct doses, because organizations like WHO, TB alliances, and other stakeholders are working tirelessly to ensure accessibility of this medication by characterizing and understanding current market and distribution pathways for current and future pediatric TB child‐friendly formulations. Notably, the Stop TB Partnership′s GDF is considered the largest procurer of pediatric TB treatment and the largest supplier to national TB programs by ensuring quality pediatric TB drugs worldwide. GDF achievements between 2007 and 2013 include supplying more than 580,000 treatments to children in over 50 countries, of which 14 are among 22 high‐burden countries [66].

Before 2019, most children with TB were treated with adult medication to develop a child formulation. Fortunately, STEP‐TB introduced the pediatric fixed‐dose combinations. Two child‐friendly FDCs (RH75/50 mg and RHZ75/50/150 mg) were developed and marketed in 20 of 22 high‐burden TB countries. They were associated with reduced pediatric TB‐associated mortality. Therefore, STEP‐TB strives to make affordable FDCs and introduce them in low‐burden countries with high‐risk groups. In 2019, good results were seen in Kenya, which was the first country to introduce this formulation. It saved 2660 lives, and you can imagine what it could do if made available worldwide. STEP‐TB recognizes the need for child‐friendly treatment for drug‐resistant TB [67].

The first pharmaceutical cost analysis in children under 15 years was done in South Africa, India, and the Philippines to assess all available adult and child formulations in RR‐TB. The cost of TB medication was influenced by local guidelines, prescribing choices, procurement practices, the prevalence of fluoroquinolone resistance, and whether RR‐TB was severe or nonsevere. Based on the results, child‐friendly formulations are more expensive than adult formulations, and dispersible tablets were found to be nine times more expensive than all solid‐dose formulations. It is important to use the GDF procurement approach to improve overall TB pricing rather than using the South African method [68].

### 5.2. Nanoformulation

The optimization study of a self‐nanoemulsifying drug delivery system using experimental design and excipients holding innate antimycobacterial activities indicated to be a good drug delivery system. The formulations were characterized for optical clarity, zone of inhibition, and globule size, and the results led to reassessing this delivery system using different excipients with inherent antimycobacterial activities [69].

The researchers are explorating new strategies for drug delivery using nanotechnology. Rifampicin‐nanoemulsion was developed and assessed for in vitro antimycobacterial activity. The liquid rifampicin‐nanoemulsion 500 mcg/mL formulation was developed using hot solvent diffusion methods with an average size of 25 nm. The encapsulated rifampicin had the same minimum inhibitory concentration as the free rifampicin. Therefore, the rifampicin nanoemulsion could be a promising alternative for oral child‐friendly pharmaceutical formulation [70].

The study has investigated the use of SEDDS as a topical route of administration as opposed to the usual improvement of lipophilic oral drugs. Cfz was used to prepare a dermal formulation using different natural oils to improve its solubility. Natural oils with high solubility were selected and used in a fixed 1:1 Smix ratio to determine the emulsifying region using a pseudoternary phase diagram. The preferred formulations were evaluated for particle size, PDI, robustness to dilution, self‐emulsification time, skin diffusion, and thermodynamic stability. The results have shown enhanced topical Cfz delivery, though some characterization might be revised to fit the dermal route of administration [71].

Most of the drugs with limited bioavailability have been formulated as microemulsions to improve solubility and intestinal absorption. Therefore, BDQ was loaded into a microemulsion to improve oral availability, which was prepared using a spontaneous emulsification method. The results indicate that the average globule size was 26.5 nm with spherical geometry and gel‐sol‐gel behavior, and it is cytocompatible at the highest 500 *μg*/mL, with higher cellular uptake than the control. Hence, there is a need to explore the use of microemulsion in the effective treatment of MDR‐TB [72].

Cfz‐SNEDDS was developed using ethyl acetate, Captex‐300, and polyethylene glycol 400. The optimized formulation has a particle size between 288.9 and 918.2 nm, zeta potential of −1.10 to −8.25, self‐emulsification time of less than 1 min, and percentage transmission at around 100%. Dissolution of 100% was achieved in 1 h, compared with < 15% for the pure drug in 1 h, and stable SNEDDS was formulated [73].

A solid SNEDDS was developed using design of experiments with caprylic acid oil, Transcutol P, and propylene glycol as surfactant/cosurfactants. The formulation was evaluated for droplet size, PDI, and percent transmission while using QTPPs and CQAs to ensure a robust formulation. The optimized SNEDDS formulation indicates a small particle size and low PDI, with morphology showing a spherical shape and a distinct boundary. It also exhibits greater drug concentration release compared to the suspension form and shows enhanced cellular internalization in cell cytotoxicity studies. The formulation remains stable for 6 months at both room temperature and freezing. Therefore, the optimized BDQ SNEDDS showed enhanced drug delivery, which is promising in MDR‐TB treatment with the potential of reduced drug frequency and drug dosage [74].

Delamanid is a novel drug approved in 2014 for MDR‐TB and has high treatment efficiency in TB, but it has drug delivery challenges due to its lipophilic nature, hindering good oral bioavailability. The development of novel formulation‐based pressurized metered dose inhalers (pMDIs) containing self‐microemulsifying delamanid mixtures challenges the idea of improving efficient delivery to the lungs. This formulation identifies the appropriate self‐microemulsifying region using ternary diagrams and using different combinations of Cremophor RH40, polyethylene glycol 400, and peppermint oil (surfactant‐to‐cosurfactant ratios 1:1, 2:1, and 3:1) and selects the most stable emulsification for further characterization. The selected formulation has shown uniform dose delivery and droplet sizes and zeta potentials of 24.74–88.99 nm and −19.27 to −10 mV, respectively. SMEDDS‐pMDI has exhibited the capability to deliver the drug to deeper areas of the lungs and showed no significant signs of toxicity at 1.56 *μg*/mL, with higher antimicrobial activity by Day 5, a minimal bactericidal concentration of 0.313 *μg*/mL, and efficient cellular uptake of the drug inside macrophage cells. Therefore, delamanid SMEDDS‐pMDI improves the effectiveness of MDR‐pulmonary TB [75].

Moxifloxacin was loaded into a mucoadhesive PEC NP system using CG and Alg. The NP formulation was to be used for ocular administration and showed good physicochemical characterization, with nontoxicity and efficacy against *P. aeruginosa* and *S. aureus* (in vitro) and BK‐induced mouse models (in vivo) [76]. A nanoemulsion of BDQ was formulated using olive oil, Tween 80, and Span 20. With a 191.6 nm droplet size, PDI of 0.1176, zeta potential of −25.9 mV, and DC of 3.15 mg/mL, it has shown potential for improved solubility, drug delivery, and administration of BDQ. Thus, further studies are needed on this formulation. The BDQ‐NE was in liquid form, making it easier for pediatric administration, providing dose flexibility based on age and weight, and allowing easier manipulation of organoleptic properties [77]. Figure 6 shows the dual‐phase release profile as expected from the nanoemulsion formulation designed for sustained drug release and improved therapeutic efficacy. There was an initial burst release of the drug with 12% release at 120 min, but it slowed down, with only 14% of the drug being released at 240 min. Therefore, there was a significant increase in release between 240 and 720 min, indicating rapid achievement of the target drug level followed by subsequent maintenance to provide prolonged treatment benefits.

**Figure 6:**
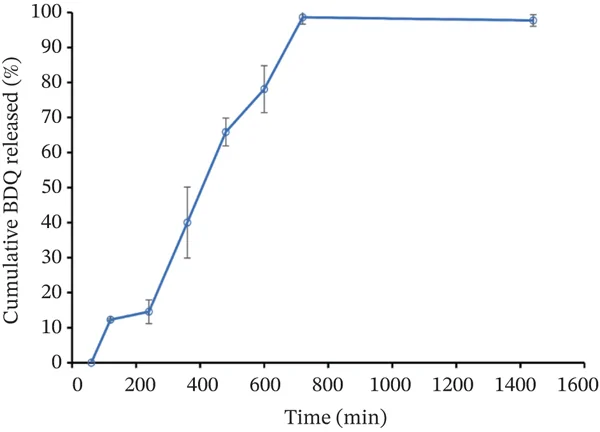
Bedaquiline release from the optimized nanoemulsion. Reproduced from [77] under a Creative Commons Attribution‐NonCommercial‐NoDerivatives 4.0 International License. The figure has been reproduced without modification.

A new drug from plant extracts has been discovered, and the form of drug delivery is yet to be determined. In vitro studies were conducted to assess the antimycobacterial activity of *Aloe vera* and *Allium sativum* against MDR‐MTB and to check their cytotoxicity and mutagenicity. The Soxhlet apparatus was used to prepare four extracts of *Aloe vera* and *Allium sativum*, and the minimum inhibitory concentration against MDR M.tb isolated from pediatric patients was determined by the BACTEC MGIT960 system. *Aloe vera* exhibits an MIC of 5–7 mg/mL, and *Allium sativum* has an MIC of 3–5 mg/mL. These extracts showed antimycobacterial activity against MDR‐MTB, with noncytotoxic and nonmutagenic properties; they can be used in patients with MDR‐MTB [78].

### 5.3. Dispersible Drug Formulation

In February 2018, levofloxacin (Lfx) 100 mg dispersible tablets were developed by Macleods Pharmaceuticals, Mumbai, India. After assessment of acceptance in children below 5 years in Cape Town, the medication was approved by the WHO and made available through the GDF. One of the approved medication was Lfx 100 mg tablets, taste masked (orange peppermint flavor), dispersible in 1 min, break line. Moxifloxacin and Lfx are bitter when crushed. To assess how the dispersible Lfx 100 mg was accepted in children, 27 caregivers were interviewed through a questionnaire; 69% found the medication to be palatable, and 81% found it easier to prepare compared to the previous adult formulation [79].

Orodispersible pharmaceutical formulation containing Z was developed and evaluated, as they are known for being stable and dissolving rapidly in the presence of saliva; hence, no chewing or drinking of water is needed. The formulation has shown rapid disintegration, drug absorption occurring within 60 min, and drug release following the Korsmeyer–Peppas mathematical model. The Z molecules have displayed 25.02 mg of drug loading; therefore, multiple orodispersible films might be needed for an average dose of 35 mg/kg based on pediatric body weight. Based on this finding, this formulation was recommended for pediatric patients below 5 years old. The formulation does not pose a choking hazard and is of microstructural quality. It is thermodynamically and environmentally stable, palatable, and easy to handle. The orodispersible formulation has the potential to solve challenges of safe administration and improve safe drug release of anti‐TB in children [80].

Cfz minitablets with a dose‐adjustable formulation were prepared via direct compression to improve oral bioavailability and have therapeutic plasma drug levels for the treatment of TB. The optimized minitablets show minimal drug release in stimulated saliva but enhance drug release in fed stimulated intestinal fluid. The result indicates the importance of bile salts in Cfz oral bioavailability, and there was no significant difference in absorption of oral suspension and minitablets. This signifies the feasibility of using low‐cost technology to address the lack of child‐friendly formulations and enhance the bioavailability of lipophilic drugs. Therefore, this process approach is suitable for formulating a CFZ suitable for children under 6 months [81].

The FlexiChew formulation of the first‐line anti‐TB isoniazid was designed as a polymeric gummy drug formulation using solid–liquid dispersion and a dependent sol‐gel processing technique. It was visually attractive and met all the characteristics of a chewable tablet. The chewable tablet encapsulated about 101.565 mg of isoniazid, which has 100% drug release in 75 min; efficient taste masking and palatability and stability studies suggest storage under controlled ambient environments, avoiding direct sunlight and without desiccants. Therefore, this type of formulation is intended to improve adherence and therapeutic efficacy in children with TB and is suitable for children above 2 years [82].

In response to close the gap of lack of child‐friendly formulation for TB, a fixed‐dose combination water‐dispersible tablet of isoniazid 50, Z 150, and rifampicin 75 mg was formulated with the lowest number of excipients according to pediatrics and fulfillment of all the pharmacopeia requirements. The developed tablets have shown disintegration in less than 3 min and friability below 1%, and they retain all APIs within 5% drug content at 6 months of storage. Therefore, the formulation developed will improve TB treatment in pediatrics because the WHO‐prequalified one has excess excipients [83].

The 3D printing (3DP) with semisolid extrusion (SSE) module for isoniazid chewable gels maintained suitable rheological properties with consistent weight, dimensions, stability, and texture, achieving a balance between printing parameters and shape maintenance with improved palatability compared to INH tablets. This innovative approach offers a promising solution for pediatric formulation development and improves acceptability [84]. As indicated in Figure 7, there is improved drug release in a chewable gel with more than 85% of the drug released within 30 min compared to the traditional isoniazid batches.

**Figure 7:**
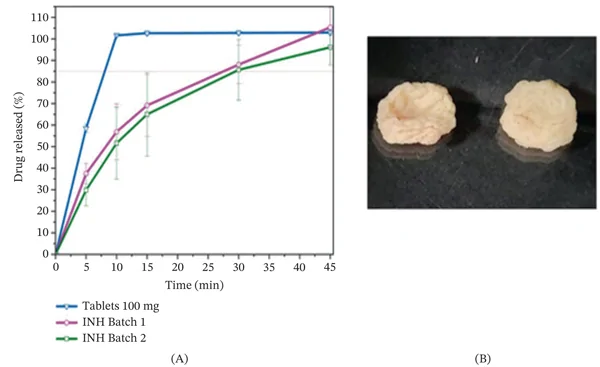
Comparing dissolution profiles between traditional tablets and 3D‐printed chewable gels of isoniazid. (A) The red line represents 85% API release, and the release in the 3D‐printed chewable gels was greater than 85% in 30 min. (B) The matrix of the chewable gel after the experiment time. Reproduced from [84] under the terms and conditions of the Creative Commons Attribution (CC BY) license.

Since the available moxifloxacin and Lzd dispersible tablet formulations have poor palatability in children, the evaluation was conducted in comparison to the newly favorable blended, more child‐friendly formulations. Children had a statistically significant preference for the novel moxifloxacin formulation over the existing dispersible tablet with suboptimal acceptability, but no clear preference for the Lzd formulation [85].

## 6. Limitations and Future Directions

### 6.1. Limitations

The research papers included were English‐language publications only, which could have excluded cultural and geographical evidence from non‐English‐speaking areas. There was also limited access to some papers, and the papers were accessible through the search engine; this could have added some evidence to the review and broadened the understanding in the field.

### 6.2. Future Directions

Therefore, future reviews should broaden to non‐English research papers, not open‐access papers only and more search engines to provide a detailed picture of pediatric TB oral treatments. There is a call to action to prioritize pediatric drug development while considering strategies to protect and care for children by collaborating with relevant stakeholders. This approach will also allow early access to novel or repurposed drugs without compromising their safety and efficiency with the formulation available [86]. Based on the literature, more research is needed to enhance these nanoformulations to improve clinical outcomes. Further clinical trials are needed to ascertain the safety and efficacy of these innovative formulations in pediatric populations. Novel pediatric formulations can ease drug administration, reduce medication errors, increase dosing acceptability, and improve medication adherence and safety.

Nanoformulations in drug delivery have made considerable advances in enhancing pharmaceutical efficacy, notably for poorly soluble medicines and those classified as BCS Class II. These techniques work together to enhance drug delivery more efficiently and effectively, improving patient outcomes and reducing adverse effects. Overall, nanoformulated MDR‐TB drugs improve children′s safety and tolerability while addressing many of the issues with conventional TB therapy. Overcoming these obstacles in current treatment is critical to improving outcomes in pediatric MDR‐TB infections. Researchers are currently striving to develop shorter, more tolerable therapy regimens, and improve treatment.

## 7. Conclusion

The treatment of pediatric TB has evolved over the years, and there are still efforts to close unmet treatment needs. It has been shown that research needs to be done in the pediatric population to have accurate PK and pharmacodynamic data, which will improve clinical outcomes. In addition to current advancements, there is a need to foster strong collaboration between pharmaceutical industries, healthcare workers, policymakers, funders, researchers, and patients/caregivers, as this will accelerate the development of optimized and suitable child‐friendly formulations. Therefore, future studies need to tap into new technologies like nanoformulation, especially for pediatric MDR‐TB treatment that lacks child‐friendly formulations distributed globally. Based on the information gathered, there is potential to explore the development of oral pediatric nanoformulations for MDR‐TB treatment.

## Author Contributions

L.N.D.: conceptualization, methodology, software, writing—original draft, writing—review and editing, and visualization. P.K.: conceptualization, writing—review and editing, validation, supervision, and project administration. Y.E.C.: conceptualization, writing—review and editing, validation, supervision, funding, and project administration. All authors agree to the final draft of the manuscript.

## Funding

The study was funded by National Research Foundation (10.13039/501100001321, PPNT230823145247).

## Conflicts of Interest

The authors declare no conflicts of interest.

## Data Availability

All data discussed in this review are derived from previously published studies, which are cited in the reference list.
